# Highlights for the Management of a Child with Hypertension

**DOI:** 10.1155/2012/364716

**Published:** 2012-08-13

**Authors:** Mouin G. Seikaly

**Affiliations:** Department of Pediatrics, University of Texas Southwestern Medical Center at Dallas, 5323 Harry Hines Boulevard, Dallas, TX 75235-9063, USA

## Abstract

Over the past several decades, childhood hypertension has undergone a considerable conceptual change, as hypertension is a predictor of future development of cardiovascular disease in adults. Childhood hypertension has distinctive features that distinguish it from hypertension in adults. Pediatric hypertension is often secondary. It is widely believed that therapeutic intervention at an early age favorably modifies the long-term outcome of hypertension. Despite its significance as a cause for morbidity, childhood hypertension is underdiagnosed and less studied with many basic issues remaining contentious.

## 1. Overview

It is widely accepted among pediatric health care providers that the risks of developing coronary artery disease (CAD) start in early life. Hypertension (HT) is a major modifiable risk factor in the development of CAD. Identification of HT at an early age may allow early intervention to prevent future end organ damage. Despite ample literature studying HT in animals and humans, our understanding of pediatric HT is still modest at best. Many questions regarding the long-term effects of antihypertensive therapy on growth and development remain unanswered. Until recently, normal blood pressure (BP) values have been scarce especially in the very young due to the relative difficulty of measuring BP in this age group [1]. The wide availability of oscillometric BP devices have made BP measurement more feasible especially in young children. Furthermore, several normative BP values are now available. Thus, the measurement of BP in infants and children at the office and hospital should now be easier and more reproducible.

### 1.1. Pathophysiology

The pathogenesis of systemic arterial hypertension is multifactorial. Hypertension is a hemodynamic manifestation of total vascular resistance (TVR), and cardiac output (CO) [2] TVR is a function blood vessel wall elasticity, myocardial contractility, and cardiac afterload. Cardiac output is the product of cardiac stroke volume (SV) and rate (HR). Both myocardial contractility and HR are regulated by sympathetic nerve activity. SV depends on myocardial contractility and preload. During the early stages of hypertension, CO is often increased. As hypertension progresses, TVR increases and CO normalizes. In a certain group of patients, hypertension develops primarily due to a decrease in the cross-sectional area of peripheral arterioles leading to an increase in resistance to flow. TVR is controlled by the interaction of vasodilators such as prostaglandins and bradykinins and vasoconstrictors such as platelet-derived growth factor (PDGF), thromboxane, and angiotensin II. Another group of patients develop hypertension due to volume over load and sodium retention. This group includes patients with renal disease, African American children, and certain genetic forms of hypertension.

### 1.2. Definition

A prevalent operational designation of hypertension is BP elevation above the 95% percentile for either age, height, or tanner stage and gender, using standardized measurement techniques on at least three separate occasions [1]. Prehypertension is defined as BP elevation between 90 and 95%. The normative sporadic BP values were updated in 2004 by The Fourth Report on the Diagnosis, Evaluation, and Treatment of High Blood Pressure in Children and Adolescents from the United States of America (US) [3]. This task force has incorporated previous data from US children and added new data from the 1999 to 2000 National Health and Nutrition Examination Survey (NHANES). Blood pressure values are based on gender, age, and height, and 50th, 90th, 95th, and 99th percentiles are provided. While this data is accepted and used worldwide normative data from other regions of the world is available. Some regional normative data significantly vary from US data, stressing the ethnic variability in BP. European data are 3–6 mmHg higher than US data [4]. In 2003 the Joint National Committee on Prevention, Detection, Evaluation, and Treatment of High BP (JNC7) further stratified hypertension in adults into prehypertension and 2 stages [5]. In 2004, The Fourth Report recommended childhood classification [6]. Stage 1 HTN is defined as being from the 95th to the 99th percentile plus 5 mmHg. Stage 2 HTN is 5 mmHg or more above the 100th percentile and represents a level of BP that requires prompt evaluation. 

### 1.3. Prevalence

The prevalence of hypertension varies among certain subgroups. The definition hypertension implies that around 5% of the general population is hypertensive. Actual BP measurement among 10 19-year-old school children, shows that the prevalence of hypertension is close to the predicted rate at 4.5%. This makes hypertension one of the most common preventable disorders facing pediatricians. Risk factors associated with hypertension include gender (males), ethnicity (African Americans and Hispanics), and increased BMI can increase the prevalence of hypertension among certain high risk groups. The prevalence of hypertension among obese children is much higher estimated to be as high as 11% [7]. Children with hypertension have 2.5 times the risk of becoming adults with hypertension. From these studies one may conclude that prevention of risk factors for the development of hypertension, such as obesity, may delay or prevent adult hypertension.

## 2. Clinical Presentation

 Symptoms of hypertension in childhood can vary depending upon the severity and duration of hypertension. Mild to moderate hypertension is often asymptomatic, while severe hypertension can present with encephalopathy and acute loss of vision (posterior reversible encephalopathy syndrome, PRES).

### 2.1. Past Medical History

Determining the duration of hypertension at presentation is of clinical consequence as it helps narrow down the list of differential diagnosis. Establishing the duration of hypertension starts by obtaining a comprehensive history. Such interview should focus on symptoms associated with hypertension such as poor sense of well-being, poor sleep, restlessness, poor growth, nose bleed, all with the potential to suggest chronic hypertension. Frequent headaches, blurred vision, chest pain, symptoms of congestive heart failure, and encephalopathy seizure all could point to an acute onset of hypertension. 

As most clinical conditions in pediatrics, etiology of hypertension is age specific, Table 1, as we and others have previously shown [8]. As such, history taking should be focused depending on the age of the child. Neonatal period, prematurity low birth weight, prolonged oxygen therapy, and history of umbilical artery catheters may provide clues as to the etiology of hypertension. Also history of urinary tract infection in infancy may predispose to renal scars and may suggest renal anomalies. In older children glomerulonephritis and in adolescent females, history of urinary tact infections or dysfunctional voiding may suggest a cause for hypertension. Symptoms of systemic illness also could include pallor, flushing, joint pains, rash, edema, gross hematuria, excessive weight gain or loss, or decreased height growth which may suggest vasculitis or glomerulonephritis. Triad of flushing, palpitations and, hypertension are often suggestive of pheochromocytoma, a rare cause of hypertension in this age group. In adolescents history is often nonspecific as the prevalence of idiopathic hypertension often increases. History should question the occurrence of headaches, sleep disturbance, visual symptoms, nosebleeds, palpitations, and episodic rapid pulse. Sleep disorder, snoring fatigue could be associated with obstructive sleep apnea, a condition overlooked in older children.

### 2.2. Dietary and Medication History

Detailed dietary history is important when deciphering the etiology of hypertension. Excessive intake of sodium or caffeinated beverages, and energy drinks is associated with hypertension. A medication history should include specific questions about over-the-counter drugs like pseudoephedrine or herbal preparations like ephedra, St. John's Wort, or licorice as well as prescription drugs. Adolescents should be questioned in private to obtain a history of substance abuse or the possibility of pregnancy. History of current or recent prescription medications such as decongestants, corticosteroids, and nonsteroidal anti-inflammatory could all suggest a cause for hypertension. 

### 2.3. Physical Exam

BP is a variable that depends on many factors including anxiety. Office hypertension also known as white coat effect is not an uncommon cause of referral for evaluation to the specialist. Studies have shown that repeated BP measurement can lower the incidence of office hypertension. A complete physical exam should focus on signs associated with the disease process that caused hypertension and signs of end organ damage associated with hypertension. 

The prevalence of secondary hypertension is high in children. An infant with hypertension abdominal mass could suggest congenital kidney disease, and pulmonary findings could suggest bronchopulmonary dysplasia. In older children, presence of edema or rash could suggest glomerulonephritis or vasculitis. Four extremities BP check is an essential part of a physical exam of a child with hypertension to evaluate for coarctation of the aorta. Café-au-lait spots could suggest neurofibromatosis often associated with hypertension either due to pheochromotocytoma or renal artery stenosis. Signs of CV disease as a complication of hypertension include gallop, tachycardia, rales, decreased breath sounds, and so forth. In severe hypertension, lethargy, loss of vision (PRES), and signs of stroke are all signs of hypertension. Signs of excessive steroids such as Cushing syndrome, for example, truncal obesity, buffalo hump, round moon faces, and hirsutism. Height and weight to calculate BMI is an essential part of the physical exam when evaluating a child with hypertension. A high BMI points to obesity as a possible cause for hypertension.

## 3. Management

### 3.1. Work-Up

Basic laboratory tests including basic chemistries, CBC, urinalysis, and renal sonogram are what the practioner should request in children who have stage 1 hypertension; please refer to Table 3 for details. If the child is symptomatic or show signs of end organ damage or symptoms of secondary causes of hypertension the GP should promptly refer the child to the specialist. End organ damage are often rare in children but can include concentric hypertrophy of left ventricular, proteinuria, microalbuminuria, and retinopathy. The younger was the age at presentation with hypertension, the higher are the chances that we find its secondary cause [5].  Table 4 lists some of the biochemical and imaging tests often recommended to evaluate a child with hypertension. 

### 3.2. Treatment

Therapeutic objectives for treating hypertension in children are listed in Table 2. Pharmacological therapy when employed should aim to control elevated blood pressure with the lowest dose and minimal number of drugs, thus minimizing potential toxicity, expense and simplifying the therapeutic regimen. The level to which elevated blood pressure is to be lowered in children and adults remains an arbitrary clinical decision. In adults, the relationship between diastolic blood pressure and the risk of cardiovascular mortality appears to be J-shaped, that is, the risk of developing cardiovascular mortality declines with lowering diastolic blood pressure up to a nadir beyond which further drop in blood pressure will increase morbidity. It is often desired to drop diastolic blood pressure to levels between 80 and 90th percentiles for age.

Current treatment recommendations are currently based on epidemiological data rather than outcome measures. There are two accepted modalities to treat hypertension in pediatrics, namely, nonpharmacological and drug therapies. The type of therapy used often depends on the age of onset, duration, and the severity of HT. It is generally accepted that borderline hypertension (90–95th percentile for age) with no evidence of end-organ damage can be treated with nonpharmacological remedies. However, Stages 1 hypertension additional drug therapy is often required. Stage 2 hypertension the other hand (above 99th percentile) often requires admission to hospital for comprehensive management.

#### 3.2.1. Nonpharmacological Antihypertensive Therapy

The safest therapeutic intervention to manage mild HT is the use of nonpharmacological remedies. Evidence for the efficacy of this type of intervention in children is not yet established. Nonpharmacological intervention has traditionally been focused on the reduction in dietary sodium intake along with high potassium (if there is no clinical contraindication) and on weight loss when the patient is obese. Obesity in children and young adults can predispose to higher BP. While there are no strong evidence in children about the effect of avoidance of tobacco, alcohol, and stress on BP control, these are desirable practices should be promoted through pediatric counseling. 

#### 3.2.2. Drug Therapy

It should be instituted whenever HT is severe or when nonpharmacological intervention alone fails to control the BP in mild to moderate HT. Major questions with regard to the long-term effects of antihypertensive drug treatment on growth and cognitive function in children remain unresolved. In addition, the absence of adequate information regarding drug interaction, volume of distribution, protein binding, metabolic degradation, and renal and hepatic excretion introduces additional concerns when treating HT in children. As such most antihypertensive drugs, however, carry a disclaimer relating to their use in children. A large number of antihypertensive agents with various sites and mechanisms of action are commercially available. Each drug has undergone extensive evaluation in adult volunteers in pre- and postmarketing clinical trials. 

#### 3.2.3. Individualized Therapeutic Regimen

The availability of newer drugs allows us to make a rational choice of antihypertensive therapy. The first step in the treatment of hypertension involves a small dose of a single drug, usually a diuretic, the dose is increased until BP goals are achieved, side effects appear, or a maximum dosage is reached. If the BP is not controlled, in spite of adequate compliance, a second and even a third drug is then added. Recently, a different approach to antihypertensive treatment has become increasingly popular where therapy is “individualized.” While vasodilators can lower high BP of almost any etiology, understanding pathophysiologic mechanisms leading to the BP elevation helps in selecting targeted therapy aimed at better control of HT. Table 3 provides guidelines for the selection of antihypertensive drugs based on our knowledge of the pathogenesis of HT in an individual child. Using these guidelines, we often initiate therapy with a calcium channel blocker agent, an ACE-I or a beta-adrenergic antagonist. These drugs are available in once-a-day dosage and have few side effects, both features reflecting positively on adherence. If monotherapy with angiotensin converting enzyme inhibitors, beta-blockers or calcium channel blockers fail to correct BP within two weeks, a diuretic or a mild vasodilator like hydralazine or prazosin are often second line therapy. A final step is to use a potent vasodilator like minoxidil or a centrally acting agent like clonidine. Often, combination therapy from different antihypertensive classes is required to achieve control of BP. Treatment of severe HT, on the other hand, requires admission to the hospital for frequent BP monitoring. For more detailed review of medications used for treatment of hypertension, the reader is referred to [4].

## 4. Long-Term Followup 

While treatment's potential to alter long-term outcome of hypertension in children sounds intuitive, clear evidence is lacking. Furthermore, persistence of hypertension into adulthood (tracking) is unknown. What is certain is that children with hypertension require frequent monitoring for end-organ damage from hypertension as well as the potential complication of antihypertensive therapy.

## 5. Summary from a General Practioner's Perspective

Hypertension is one of the most common preventable disorders facing pediatricians. Risk factors associated with hypertension include gender, ethnicity, and BMI. Adult hypertension correlates with childhood BP. It is then rather instinctive that prevention of risk factors associated with hypertension in childhood, such as obesity, may delay or prevent adult hypertension. Furthermore, the development of cardiovascular disease and renal disease later in life is also suspected to be associated with childhood hypertension. Hence, it cannot be overemphasized that early detection of hypertension by routine measurement of BP is an essential part of any office visit.

## Figures and Tables

**Table 1:** Causes of hypertension in children by age group (percentage).

Diagnosis		Age group				Total
	0–2 m	2 m–1 yr	2–6 yrs	7–11 yrs	12–18 yrs	0–18 yrs
Renal disease	83	56	83	70	56	67
Primary hypertension	0	11	14	30	35	23
Others	17	33	4	0	9	10

Adapted from [7].

**Table 2:** Therapeutic objectives for treating hypertension in children.

Achieve a diastolic blood pressure <85th percentile for children of same sex, chronological age, and body mass.	
Control hypertension with nonpharmacological means when possible.	
Use the smallest number of antihypertensive drugs and the lowest dose of each drug necessary for consistent blood pressure control and minimal drug side effects.	
Design treatment programs that are consistent with maximum likelihood of patients compliance.	
Achieve long-term prevention of end-organ damage and promote normal growth and development.	

**Table 3:** Drug options for initial therapy for hypertension in children.

Class of drugs	Patients' characteristics
Diuretics	Volume-overload, low plasma renin activity, black race, oral contraceptive therapy, and congestive heart failure.
Angiotensin converting inhibitors/angiotensin receptor blockers	High plasma renin activity, unilateral renovascular hypertension, renal insufficiency, glomerular proteinuria, congestive heart failure, diabetes mellitus, gout, and hyperlipidemia.
Calcium channel blockers	Emergency hypertension, black race, diabetes mellitus, chronic obstructive lung disease, bronchopulmonary dysplasia, gout, hyperlipidemia, and peripheral vascular disease.
Beta-adrenergic antagonists	Contracted intravascular volume, high plasma renin activity, attention deficit disorder, hyperdynamic circulation, anxiety, migraine, steroid intake, hyperthyroidism, and neuroadrenergic tumors.

**Table 4:** Suggested work-up for stages 1 and 2 hypertension.

	Test	To evaluate for
Blood and urine	(A) Complete blood count; blood urea nitrogen, electrolytes, calcium phosphorous, and albumin	(A) Renal function
	(B) Plasma Renin	(B) Renovascular HT
	(C) Complements 3 and 4; ANA, antinuclear antibody; anti-DNA and antidouble-stranded desoxynucleic acid antibody	(C) Glomerulonephritis
	(D) Antineutrophil cytoplasmic antibody (ANCA); anti-GBM and antiglomerular basement membrane antibody	(D) Vasculitis
	(E) Thyroxine, T4; thyroid stimulating hormone, TSH; adrenocorticotrophic hormone, ACTH; OH, hydroxy; deoxycorticosterone, DOC; parathyroid hormone, PTH	(E) Hormonal
	(F) Serum and urinary catecholamine and metanephrines	(F) Neurogenic tumors

Imaging	(A) Renal ultrasound	(A) Renal malformation, medical renal disease, and renal scars
	(B) Mercaptoacetyltriglycine and MAG 3 scan with or without furosemide. With or without Captopril	(B) Obstuctive uropathy, renovascular HT, and differential renal function
	(C) Dimercaptosuccinic acid, DMSA	(C) Vesicoureteral reflux and reflux nephropathy; differential renal function
	(D) Voiding cystourethrogram, VCUG; digital subtraction angiography, DSA	(D) Vesicouretheral reflux and structural bladder abnormalities
	(E) Magnetic resonance angiography, MRA; digital subtraction angiography, DSA; computed tomographic angiography, CTA; magnetic resonance angiography, MRA	(E) Renovascular
	(F) MIBG, metaiodobenzylguanidine	(F) Pheochromocytoma

Tests of end organ damage	(A) Echocardiogram, CXR ECG	(A) Cardiovascular morbidity
	(B) Urinalysis	(B) Proteinuria
	(C) Microalbuminuria	(C) Glomerular hyperfiltration
	(D) Ambulatory BP monitoring	(D) Absence of diurnal rhythm and white coat effect
